# Upon the Treatment of Tabes Dorsalis, by Means of Uninterrupted Galvanic Currents

**Published:** 1858-07

**Authors:** R. Remak


### ARTICLE IX.

[TRANSLATED BY E. L. HOLMES, M. D.]

### UPON THE TREATMENT OF TABES DORSALIS, BY MEANS OF UNINTERRUPTED GALVANIC CURRENTS.—BY R. REMAK.

(From the records of a meeting of the Hufeland Society, held March 26, 1858.)

Mr. Remak next offered a few suggestions regarding the origin and development of the experiments in which he had been engaged since July, 1856, in the treatment of tabes dorsalis. The idea was derived from the knowledge of the fact, that the *continued* application of uninterrupted galvanic currents passed through the sciatic nerves of patients, who experienced symptoms of the so-called tabes dorsalis, had the effect to increase their steadiness of step, which was shown by their increased power to walk and stand with eyes closed. A decided beneficial effect was also produced upon accompanying nervous weakness of bladder and rectum. Comparative experiments upon a large number of patients, during the winter of 1856–57, showed that the good effects of the method above proposed was limited to certain degrees of development and to particular species of this multiform disease. The fact was noticed in May, 1857, and first reported in the *Deutsche Klinik* (1857, No. 50), that the interrupted application of unbroken currents operated powerfully in increasing the action of the muscles in diseases of the nervous centres, which the uninterrupted passage of unbroken currents or the use of broken currents did not produce. This discovery also (in the summer of 1857) rendered the treatment of those forms of tabes dorsalis less difficult, in which the muscles manifest an evident complication.

An episode was introduced in the history of these inquiries by the discovery, already mentioned (*Deutsche Klinik*, 1857, No. 50), but not fully investigated, according to which the stimulation of the nerves of the arm was found to exert a decided influence upon the central organ, and also to restore steadiness to the step of the patient. The use of an unbroken current has received a wider application since the autumn of 1857, in the scientific treatment of the spinal cord itself. A physiological analysis of the clinical experiments has already induced the belief that the atrophy of the spinal cord, from which the disease derives its name, in many instances, is only the result of a chronic exudative inflammation in the cellular portion of the substance of the spinal cord. This opinion was founded upon the remarkable pathologico-anatomical investigations of Rokitansky, reported to the Imperial Academy of Science of Vienna ("Hypertrophy of the Cellular Tissue in the Nervous System," from the reports of the Math. Phil. Class of the Academy of Science, May, 1857), the subject of which has been more definitely discussed by Mr. Remak. Moreover, from a comparison of a large number of experiments upon the influence of uninterrupted currents in exudative inflammation of the cellular tissues, especially in the joints, Mr. Remak has become convinced that the "electrolytic influence" of unbroken currents, already mentioned (*Deutsche Klinik*, 1857, No. 50), so far as absorption and dilatation of the vessels are concerned, probably deserves the appellation of an antiphlogistic remedy in certain inflammatory conditions, in which the use of depletion, mercurials, iodine, etc., would be in part if not wholly contra-indicated. This opinion was supported by examples, which were also employed to illustrate the benefit of unbroken currents in surgery.

As examples of the direct influence of the unbroken currents upon probable inflammatory and infiltrated condition of the substance of the spinal cord, three cases were reported. The first was of a man, forty years of age, who had for three years suffered from a decided tabes dorsalis, complicated with militurea and paralysis of the bladder. After thirty-five applications of the galvanic currents to the legs and the cauda equina, the paralysis of the bladder during the day (but not during the night) gradually disappeared; the tormenting thirst and the increased secretion of urine were removed, and the sugar in the urine (by Fehring's test) was no longer found. The application of the current to the lower four vertebræ, where the patient experienced pain, gave him steadiness of step, and the increasing power to stand with the eyes closed. After sixty-five applications (from November 20, 1857, to February 6, 1858), the patient was dismissed in an improved state of health, with directions to return in the summer, since the paralysis of the bladder at night still remained. Two other patients with the same disease were shown to the society. The first, a shoemaker, thirty years of age, from the district of the factory physician, Dr. Neithard, was attacked in June, 1857, with a paralysis of the legs and rectum. While in an hospital, the paralysis of the legs had improved, but not that of the rectum. The use of "inductive" currents, according to the method of Duchenne

(out of the hospital), had been without benefit. The patient has been treated twenty-five times since January 31. with the galvanic currents; after the first five applications, the patient's step became firmer and the rectum in a better condition. At present, the patient is only troubled by an inaction of the right gluteal muscles, which prevent him from long retaining a sitting posture; although, in this respect, the improvement is continually progressing. The treatment of the lower portion of the spinal cord, although the patient had experienced slight pain in this region, which passed away after the first applications, exhibited the most visible effects even upon the disease of the rectum. The treatment of the muscles offered great difficulties, since, in consequence of the use of electricity, a great degree of muscular contraction was present.

In regard to the effect of "inductive" currents, Mr. Remak referred to his essay in the *Deutsche Klinik*, No. 2.

The second patient, a cooper, thirty years of age, also from the district of Dr. Neithard, was attacked in April, 1857, with a complete paralysis of the bladder, and in July of the same year of partial paralysis of both legs, which, during a residence of seven weeks in the hospital, became evidently worse, as the patient maintained, in consequence of the application of cups to the back. He was afterwards for six weeks electricised by the same physician as the first patient, with such bad success, that he was obliged to discontinue the treatment. On account of the benefit experienced by the first patient, he was placed by Dr. Neithard, on the 23d of July, under the care of Mr. Remak. Although the difficulty of walking, which the patient experienced in consequence of muscular contraction, did not correspond entirely with the symptoms of tabes dorsalis, yet the inequality of the pupils, and the unsteadiness of step on closing the eyes, left no doubt regarding the central character of the affection. After a few applications of the currents directed particularly to the spinal cord, there resulted a decided and continued improvement in the gait and especially in the paralysis of the bladder, which gave no trouble either day or night, although the stream of urine had not yet recovered its normal volume. Mr. Remak gave, in conclusion, as the result of his experience in the so-called tabes dorsalis, that, not only in those forms of it which exhibited few or no symptoms of previous inflammation, but also in those which, according to Rokitansky, are symptomatic of myelitis centralis, the earliest application possible of the unbroken currents to the spinal cord and to the nerves and muscles affected secondarily, is a means better than all others in producing a cure, or at least a palliation. Whether atrophy of the spinal cord is always, as maintained by Rokitansky, a result of myelitis, or primary in its origin, and in this case incurable, even in its early stages, Mr. Remak is unable to decide.

